# In the Midst of a Cytokine Storm—The Hemophagocytic Syndrome (Hemophagocytic Lymphohistiocytosis/Macrophage Activation Syndrome)

**DOI:** 10.1111/vcp.70155

**Published:** 2026-10-19

**Authors:** Joanne B. Messick

**Affiliations:** ^1^ College of Veterinary Medicine Purdue University West Lafayette Indiana USA

## Abstract

**Background:**

Hemophagocytic syndrome (HPS)/hemophagocytic lymphohistiocytosis (HLH) is a life‐threatening hyperinflammatory syndrome characterized by immune dysregulation, cytokine overproduction, hemophagocytosis, cytopenias, and multiorgan dysfunction. Although diagnostic criteria are established in human medicine, standardized criteria and validated biomarkers are lacking in dogs and cats.

**Objective:**

To review the pathogenesis, clinical and clinicopathologic features, diagnosis, and differential diagnoses of HPS/HLH, with emphasis on dogs and cats, and to propose a framework for establishing standardized diagnostic criteria for canine HPS.

**Methods:**

Human and veterinary literature on HLH, HPS, and macrophage activation syndrome was reviewed, including diagnostic criteria, biomarkers, bone marrow morphology, immunohistochemistry, flow cytometry, and molecular diagnostics. Representative canine cases of secondary HPS and hemophagocytic histiocytic sarcoma were evaluated to illustrate diagnostic challenges.

**Results:**

HPS results from dysregulated activation of cytotoxic lymphocytes and macrophages, with interferon‐γ playing a central role in sustaining hyperinflammation. In dogs and cats, HPS is most commonly associated with infections, neoplasia, and immune‐mediated disease. Clinical and laboratory findings are nonspecific, and although hemophagocytosis supports the diagnosis, it is neither required nor sufficient to establish HPS. Distinguishing reactive HPS from histiocytic neoplasia and other non‐neoplastic causes of hemophagocytic activity—including drug‐associated changes, post‐transfusion hemophagocytosis, and PIMA‐associated histiocytic activity—requires integration of cytomorphology, tissue distribution, immunophenotyping, and molecular diagnostics. Human HLH‐2004 criteria and the H‐score provide useful diagnostic frameworks, but neither has been validated for veterinary species.

**Conclusions:**

HPS is a challenging and potentially fatal syndrome in dogs and cats, which requires comprehensive clinicopathologic evaluation. Prospective studies are needed to establish species‐specific diagnostic criteria and validate quantitative morphologic, immunophenotypic, molecular, and biomarker‐based approaches to distinguish HPS from infectious, immune‐mediated, and neoplastic disorders in which phagocytic activity is prominent.

## 1 Introduction

### 1.1 Overlapping Terminology

Hemophagocytic lymphohistiocytosis (HLH) is the term commonly used to describe a syndrome in human patients characterized by overwhelming systemic inflammation [1]. HLH encompasses both familial and secondary forms of this syndrome, highlighting the involvement of lymphocytes and histiocytes (macrophages) in its pathogenesis. Macrophage activation syndrome (MAS) is a subset of HLH, primarily associated with autoimmune diseases such as systemic juvenile idiopathic arthritis and systemic lupus erythematosus (SLE). MAS focuses on the pivotal role of macrophage overactivation in driving the disease [2]. The term “cytokine storm” is often used more broadly to describe the hyperinflammatory state driven by excessive production of proinflammatory cytokines, a defining feature of HLH [3]. However, other conditions like sepsis, severe infections, and certain cancers may also secrete high levels of these cytokines [4]. Hemophagocytic syndrome (HPS) is the term most used in veterinary medicine to describe this condition and emphasizes the finding of hemophagocytosis, where activated macrophages engulf blood cells, a phenomenon observed in bone marrow or other tissues [5, 6]. Although these terms overlap, each emphasizes specific aspects of a syndrome characterized by severe immune dysregulation. To maintain consistency, this review uses the term “HPS” throughout when referring to the condition in general and when discussing the syndrome in dogs and cats. The term “HLH” is reserved for discussions specific to the human syndrome, with contextual references provided as needed.

### 1.2 Hemophagocytic Syndrome (HPS)

HPS is an uncommon but potentially life‐threatening immune disorder that arises from excessive activation of the immune system [7]. The syndrome is often described as a paradoxical immune reaction characterized by a hyperactive but ineffective immune response. Cytotoxic T lymphocytes (CTLs) and natural killer (NK) cells are critical components of the immune response, with complementary roles played in identifying and ultimately eliminating infected or abnormal cells. However, in HPS, these cells fail to destroy their target and become dysregulated, leading to an exaggerated inflammatory response. A key feature of HPS is the activation of macrophages, which indiscriminately engulf and destroy various blood cells, including erythrocytes, leukocytes, and platelets. As a result, patients often present with symptoms such as fever, splenomegaly, cytopenias, and dysfunction of multiple organs [8, 9]. Understanding HPS is crucial, as early recognition and management can greatly influence patient outcomes. This review explores the complexities of HPS, focusing on its pathophysiology, potential triggers, clinical manifestations, and the diagnostic challenges encountered, to highlight differential diagnoses that need to be carefully considered and excluded.

## 2 Pathophysiology and Pathogenesis of HPS

### 2.1 Dysregulation of Cytotoxic T Lymphocytes, Natural Killer Cells, and Macrophages

Under normal physiologic conditions, when cytotoxic CD8+ T lymphocytes or NK cells encounter an infected cell, tumor cell, or other abnormal cells (target cells), they release cytolytic granules containing two key proteins: perforin and granzymes [10]. Perforin forms pores in the target cell's membrane, allowing granzymes to enter and destabilize the cell, ultimately triggering apoptosis. For this process to function effectively, both perforin and granzymes must be structurally intact, correctly trafficked within the cytotoxic cell, and properly packaged into granules. These granules then undergo exocytosis at the immunologic synapse—the interface between the cytotoxic cell and the target—ensuring their contents are delivered into the target cell to promote its destruction [11]. Normally, perforin and granzymes facilitate the destruction of target cells, eliminating the immune‐activating stimulus. This reduction in antigen stimulation of cytotoxic immune cells initiates their apoptosis, a crucial mechanism for downregulating the immune response. However, the antigenic stimulus remains unresolved in HPS, causing the immune response to intensify, with activated immune cells continuing to proliferate and release proinflammatory cytokines [12]. The secreted cytokines activate macrophages, triggering hemophagocytosis and the release of additional inflammatory cytokines by the macrophages. This cascade causes systemic inflammation, extensive tissue damage, and organ dysfunction or failure, which define the clinical and laboratory features of HPS [13].

### 2.2 Cytokine Storm

In HPS, overactivated immune cells, including CD8+ T cells, NK cells, and macrophages, produce excessive amounts of proinflammatory cytokines, which result in a phenomenon known as a cytokine storm [4, 14, 15, 16]. This uncontrolled release of cytokines, driven by the dysregulated immune response, is often triggered by infections, malignancies, autoimmune disorders, or genetic mutations (Figure 1). The cytokine storm creates a self‐sustaining cycle of inflammation, immune overactivation, and widespread tissue damage.

**FIGURE 1:**
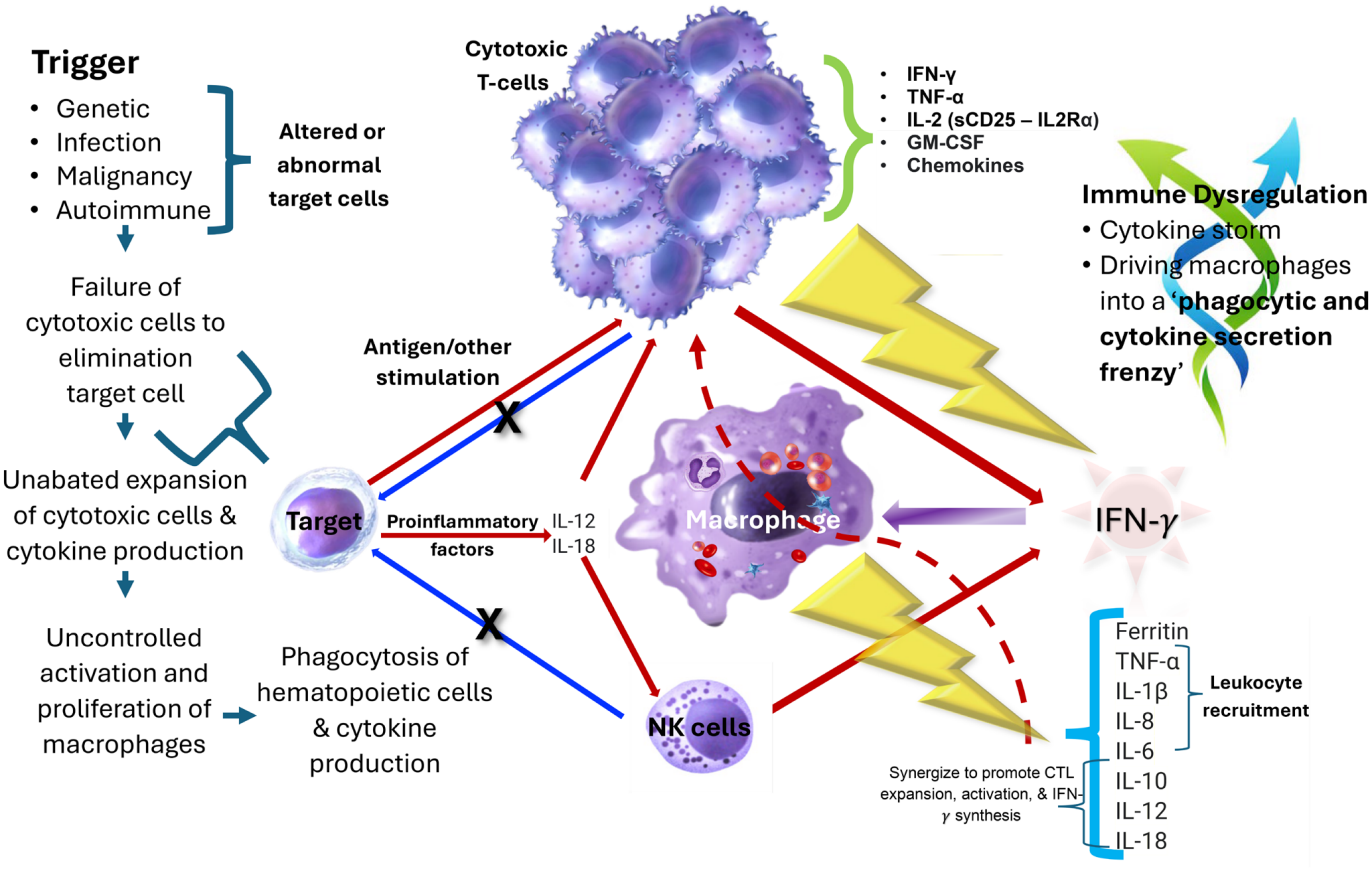
Hemophagocytic syndrome (HPS) is a hyperinflammatory condition resulting from dysregulated immune activation. Beginning at the top left, immune activation is initiated in response to altered or abnormal target cells (trigger); however, defective cytotoxic function (CTL and/or NK cells) prevents effective target cell elimination (solid blue lines). Persistent antigenic stimulation results in sustained expansion of CTLs and excessive cytokine production (green brackets), with IFN‐γ highlighted as a key mediator (upper yellow lightning bolt). These cytokines drive uncontrolled activation and proliferation of macrophages (purple line), particularly within the bone marrow, where they phagocytize hematopoietic cells and release additional pro‐inflammatory mediators (lower yellow lightning bolt). This self‐amplifying loop (solid red lines) culminates in a systemic cytokine storm and hyperinflammatory state, with macrophage‐derived cytokines further synergizing (dotted red line) to promote continued CTL expansion, activation, and IFN‐γ synthesis. CTL, cytotoxic T lymphocytes; GM‐CSF, granulocyte‐macrophage colony stimulating factor; IFN‐γ, interferon‐gamma; IL, interleukin; NK, natural killer; sCD25—IL2R‐α, soluble CD25—interleukin‐2 receptor‐alpha; TNF‐α, tumor necrosis factor‐α.

Patients with HPS exhibit markedly elevated levels of various inflammatory cytokines in the serum, including interleukins (IL‐1β, IL‐2, IL‐6, IL‐12, IL‐16, IL‐18), tumor necrosis factor‐alpha (TNF‐α), and interferon‐gamma (IFN‐γ) [16]. Increases in the soluble interleukin‐2 receptor (sIL‐2R), also known as soluble CD25 (sCD25), are frequently detected. These levels increase due to the shedding of the membrane‐bound receptor from activated T lymphocytes into the bloodstream. Persistently high or rising levels of sIL‐2R in a patient with HPS may indicate severe disease activity, poor prognosis, or an inadequate response to treatment [17]. However, it is important to note that this finding is not specific to HPS, and high levels can also occur in other conditions, including various lymphoproliferative disorders [17].

Among the elevated cytokines in HPS, IFN‐γ plays a central role in driving disease progression [18, 19]. Elevated levels of IFN‐γ stimulate macrophages, enhancing their phagocytic activity and triggering the release of additional proinflammatory cytokines. This escalation fuels a self‐reinforcing cycle of inflammation and immune system dysregulation. Experimental models of HPS, such as perforin‐deficient mice infected with lymphocytic choriomeningitis virus, have demonstrated that both CD8+ T lymphocytes and IFN‐γ are crucial for disease development [20]. These findings emphasize the critical role of IFN‐γ in HPS pathogenesis and highlight its potential as a key therapeutic target to improve patient outcomes [20, 21].

## 3 Classification and Epidemiology of HLH

### 3.1 Classification and Blurred Distinctions

In humans, HLH is categorized into two forms: primary (familial) and secondary (acquired) [7, 13]. Although both forms share overlapping clinical and pathologic features, their underlying causes and associated conditions differ. The familial form of this syndrome is due to defects in cytotoxic lymphocyte function. It is caused by mutations in genes that regulate the trafficking, exocytosis, or function of cytolytic granules, such as PRF1 (encoding perforin), UNC13D (encoding Munc13‐4), STX11, and STXBP2 [22, 23]. These mutations result in loss of protein function and impaired ability of NK cells and CD8+ cytotoxic T lymphocytes to eliminate antigenic stimuli effectively, leading to persistent immune activation. Typically, the familial form of HLH presents in infancy or early childhood and is characterized by fever, cytopenias, hepatosplenomegaly, hemophagocytosis, and organ dysfunction [24]. There are no reports of an inherited form of HPS in dogs and cats.

Unlike primary or familial HLH (fHLH), which is caused by a genetic mutation, secondary HLH (sHLH) is often associated with infections, malignancies, autoimmune diseases, or other systemic disorders. Only the secondary form of this syndrome is recognized in dogs and cats. Viral infections, particularly Epstein–Barr virus (EBV) and COVID‐19 in humans, are common infectious triggers, although bacterial, fungal, and parasitic pathogens can also induce the syndrome in both human and veterinary patients [6, 7, 25]. Bacterial infections frequently linked to HLH in humans include those caused by rickettsial and mycobacterial organisms [9], whereas veterinary cases associated with *Mycoplasma haemofelis* , *Ehrlichia canis* , and *Borrelia burgdorferi* infections have been reported [26]. Nevertheless, a wide range of other bacterial pathogens also have been implicated. Endemic dimorphic fungi, such as *Histoplasma* and *Blastomyces*, have been identified as triggers for this syndrome in both humans and dogs [6, 7]. Parasitic infections linked to this syndrome most often involve *Plasmodium* (malaria), *Leishmania* (leishmaniasis), and *Babesia* (babesiosis) species [7, 25]. In malignancy‐associated HLH, hematologic cancers such as lymphoma or leukemia are frequent culprits [8, 27, 28, 29, 30, 31], Autoimmune conditions, including systemic lupus erythematosus and immune‐mediated hemolytic anemia (IMHA), can also precipitate the syndrome in human patients [8]. Additionally, certain medications or immunotherapies that alter immune responses have been implicated in humans [13, 32]. Prompt identification of the underlying etiology is essential for targeted treatment and improving patient outcomes.

The distinctions between familial and secondary HLH are increasingly recognized as overlapping rather than strictly separate [9]. There are reports of children with fHLH that remain asymptomatic until an external trigger initiates the hyperinflammatory response [33]. Similarly, individuals with sHLH may have underlying genetic predispositions or polymorphisms that impair immune regulation, even if they lack classic mutations associated with fHLH. Advances in genetic testing have revealed that a significant subset of sHLH cases may have partial defects (monoallelic mutations) in the same pathways implicated in fHLH, suggesting that these conditions exist on a spectrum [34]. This overlap highlights the importance of considering both genetic and environmental factors when diagnosing and managing HLH. The potential genetic influence on the secondary form of this syndrome in dogs and cats has yet to be investigated.

### 3.2 Timeline for Identification and Characterization

The timeline of HLH‐related publications in the human and HPS‐related publications in veterinary literature reflects the progressive unraveling of this complex syndrome [9]. The secondary form of HLH in people was first documented in 1939 by Bodley‐Scott and Robb‐Smith, whereas in 1952, Farquhar and Claireaux described a similar condition in two siblings, now recognized as fHLH [35, 36]. Both reports highlighted characteristic features, including persistent fevers, progressive cytopenias, hepatosplenomegaly, and hemophagocytosis observed at autopsy. In subsequent decades, researchers increasingly linked HLH to triggers such as infections, malignancies, and autoimmune diseases. Nearly 50 years passed before the first cases of a similar syndrome were identified in dogs and cats (Figure 2) [5]. The diagnosis of HPS in these cases was based on the presence of cytopenias involving two or more cell lines, with at least 2% hemophagocytes observed in the bone marrow and was associated with an underlying infection or neoplasia. More recent studies in dogs and cats have investigated additional potential triggers of HPS, including lymphoma, various infectious agents (bacterial, fungal, and viral), and immune‐mediated conditions [6, 26, 27, 28, 29, 30, 31, 37, 38, 39, 40, 41, 42, 43].

**FIGURE 2:**
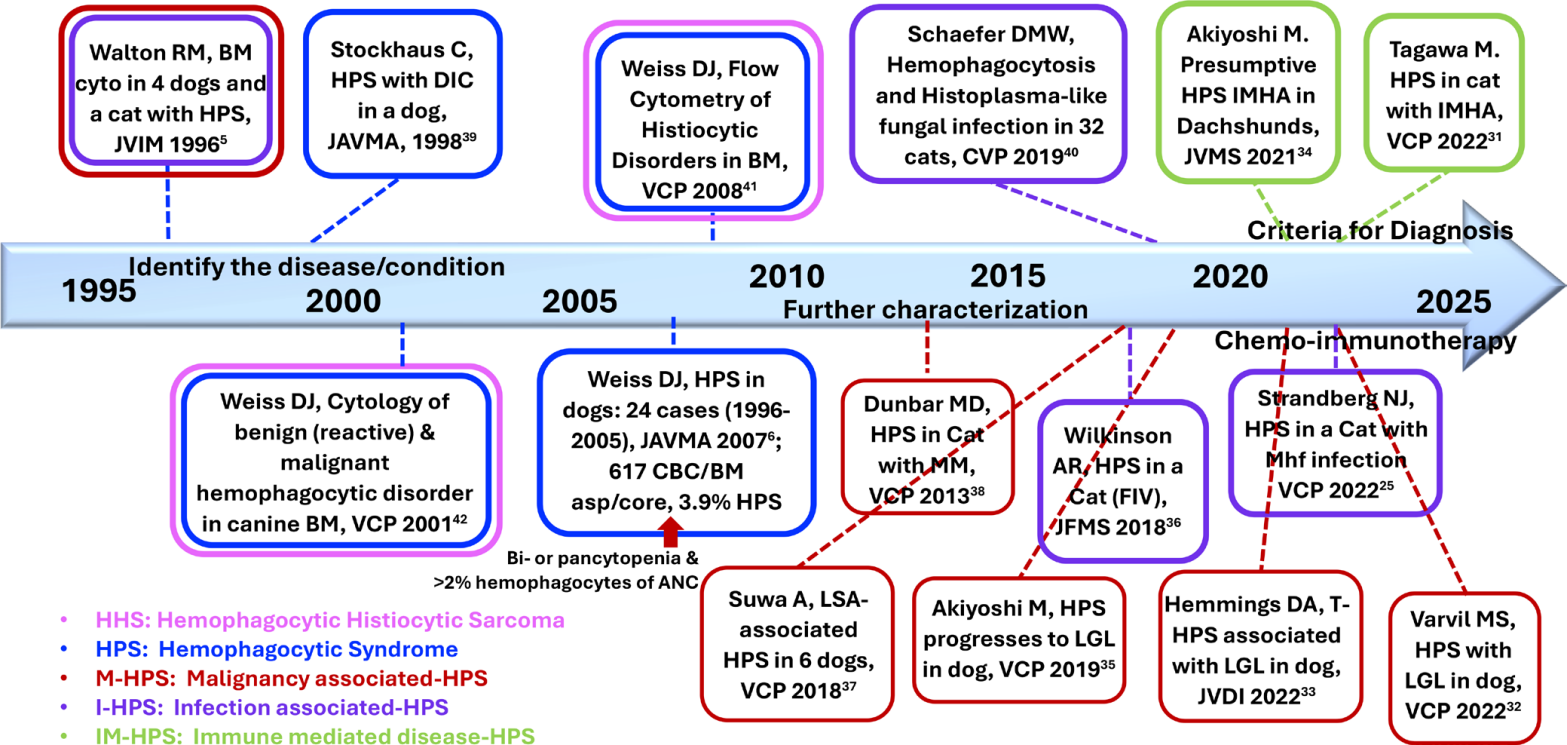
This timeline displays the main publications for hemophagocytic syndrome (HPS) in dogs and cats over the last 30 years. HPS was first described in veterinary literature in the mid‐90s (about 50 years after its first description in human literature). The criteria for its diagnosis in veterinary species rely on 2 main features: (i) bicytopenia or pancytopenia in the blood and (ii) > 2% hemophagocytic macrophages in the bone marrow aspirate. Although triggers for HPS in dogs and cats have been identified, collaborative efforts are still needed to systematically evaluate criteria for diagnosing and managing HPS in these species.

The early 2000s marked pivotal advancements, with the discovery of genetic mutations underlying fHLH and the introduction of standardized diagnostic and treatment protocols. Initial diagnostic guidelines developed by the HLH Study Group in 1994 (HLH94) included fever, splenomegaly, hemophagocytosis in the bone marrow or other tissues, cytopenias, hypertriglyceridemia, and/or hypofibrinogenemia [44]. With the HLH‐2004 protocol, these criteria were expanded to include low or absent NK cell activity, hyperferritinemia, and elevated sIL‐2R (sCD25) [25]. A treatment study also initiated by the HLH Study Group, consisting of corticosteroids and the antineoplastic etoposide, followed by hematopoietic stem cell transplantation for patients with fHLH, led to dramatic improvements in the survival of pediatric patients [45]. Since there are no prospective studies describing first‐line therapy for HLH in adults, treatment in this population is guided by pediatric data. Breakthroughs in molecular biology and immunology during the 2010s provided deeper insights into HLH's pathophysiology, particularly the roles of perforin dysfunction, CTL dysregulation, and cytokine storms [8]. More recent studies have focused on targeted therapies, including interferon‐gamma inhibitors and T cell‐mediated gene therapy [46, 47, 48]. To date, no comprehensive studies have been undertaken to evaluate the diagnostic criteria or determine effective treatments for this syndrome in dogs and cats.

### 3.3 Epidemiology

Retrospective studies conducted at major academic centers report that the incidence of HLH ranges from 1 to 225 cases per 300 000 children, with variation by country [7, 8]. The mean age at diagnosis in pediatric patients is approximately 1.8 years, and cases are distributed equally between males and females. In contrast, fewer epidemiologic studies have evaluated HLH in adults. Available data suggest that the sporadic form accounts for approximately 1 in 2000 adult admissions at tertiary medical centers [8]. The mean age at presentation in adults is approximately 50 years, with a male predominance overall. An exception is macrophage activation syndrome, in which females appear to be at increased risk. No consistent racial or ethnic predilection has been identified for either pediatric or adult HLH [8].

HPS in dogs was identified in 3.9% of bone marrow samples evaluated at one veterinary teaching hospital, with Tibetan Terriers being overrepresented [6]. In this retrospective study, the median age of affected dogs was 7.2 years (range 1–13 years), with no significant differences in age or sex compared to dogs with other bone marrow diseases. The syndrome in dogs has been linked to immune‐mediated (SLE, immune‐mediated thrombocytopenia (ITP), and IMHA), infectious (*Salmonella* spp., *E. coli* , *Blastomyces* spp., and *Borrelia* spp), and neoplastic‐myelodysplastic disorders (lymphoma, multiple myeloma, myelodysplastic syndrome, and acute myeloid leukemia), but may also occur in the absence of a known cause [6, 26, 27, 28, 29, 30, 31, 37, 38, 39, 40, 41, 42, 43]. HPS was found to be a serious disease condition, often having a poor prognosis. The outcome may depend on several factors, including the underlying cause, the timeliness and effectiveness of treatment, and the dog's overall health [6].

## 4 Presentation of HLH (Humans) and HPS (Dogs and Cats)

### 4.1 Clinical Characteristics

The clinical manifestations of HLH in humans and HPS in animals are nonspecific and can mimic other conditions, which often complicate establishing a definitive diagnosis. For example, signs and symptoms such as fever, cytopenias, and organomegaly (e.g., splenomegaly, hepatomegaly, and lymphadenopathy) in HLH can resemble those seen in systemic infections, including acute bacterial sepsis and disseminated viral or fungal infections [48]. The excessive production of proinflammatory cytokines in HLH, contributing to systemic inflammation, may lead to symptoms like rash, erythroderma, edema, and organ dysfunction (acute liver failure, acute kidney injury, and respiratory distress) [49]. Other clinical manifestations of HLH, such as gastrointestinal symptoms (diarrhea, nausea, vomiting, and abdominal pain), are nonspecific but have been reported in both humans and dogs. Neurologic symptoms, such as seizures, cranial nerve deficits, or altered mental status, are most often identified in pediatric cases and, to date, have not been recognized in association with veterinary HPS cases [50].

The clinical signs most frequently recognized in dogs with HPS are fever, jaundice, splenomegaly, hepatomegaly, and diarrhea [6]. The rarity of this syndrome in cats, with only three reported cases, precludes generalizations about clinical signs in this species [26, 37, 38]. Nevertheless, it must be emphasized that a single clinical feature alone is not diagnostic for HPS, and the overlapping clinical signs and symptoms in this syndrome underscore the need for a thorough clinical and laboratory evaluation to distinguish it from other conditions.

### 4.2 Routine Laboratory Characteristics

Laboratory data characteristic of HLH often reflect the severe immune dysregulation and systemic inflammation underlying this condition. Key findings include profound cytopenias, such as anemia, thrombocytopenia, and leukopenia, which result from bone marrow suppression and hemophagocytosis, typically involving multiple cell lines [51]. The overwhelming cytokine storm, particularly involving IFN‐γ and TNF‐α, suppresses bone marrow function, leading to ineffective hematopoiesis. Inflammatory damage to the bone marrow microenvironment further disrupts the production of blood cell precursors. Concurrently, immune‐mediated destruction of peripheral blood cells contributes to the cytopenias seen in HLH. These reductions are typically bi‐ or pancytopenic and serve as a significant diagnostic marker in conjunction with other clinical and laboratory criteria [44]. Cytopenias also reflect the severity of the hyperinflammatory state and may aid in monitoring disease progression and treatment response.

Routine serum chemistry findings in human HLH may suggest hemolysis, with hyperbilirubinemia and elevated lactate dehydrogenase (LDH) [25, 52]. Bilirubin increases due to excessive phagocytosis of red blood cells by activated macrophages, leading to heme metabolism and the release of unconjugated bilirubin. This is compounded by liver dysfunction caused by the inflammatory cytokine storm, impairing the liver's ability to process and excrete bilirubin effectively. LDH, a marker of cell turnover, rises due to widespread tissue destruction and hemolysis. Both RBC destruction and overall immune‐mediated cytotoxicity release LDH into the bloodstream. Additionally, tissue hypoxia and necrosis resulting from the hyperinflammatory state further amplify LDH levels [52]. Although these markers are not specific to HLH, their elevation in the context of other diagnostic criteria can support the diagnosis and provide insight into disease severity and tissue damage [25, 52].

### 4.3 Specialized Diagnostic Studies

#### 4.3.1 Serum Ferritin

High levels of ferritin are a prominent feature of HLH, serving as both a marker of hyperinflammation and increased red cell turnover, as well as a valuable diagnostic clue. Ferritin, an acute‐phase reactant involved in iron storage, significantly increases due to excessive cytokine production, particularly IL‐1 and TNF‐α, during systemic hyperinflammatory responses [53]. In HLH, serum ferritin levels often exceed 10 000 ng/mL, distinguishing it from other inflammatory or infectious conditions where elevations are typically less pronounced [53, 54, 55].

The diagnostic value of serum ferritin concentrations in HLH is enhanced when considered alongside other clinical and laboratory criteria, such as cytopenias, splenomegaly, and elevated sCD25 concentrations. Serial ferritin measurements can also help monitor disease activity and treatment response, with declining levels indicating therapeutic effectiveness [56]. Elevated ferritin levels in HLH reflect the syndrome's intense hyperinflammatory state, with the degree of increase correlating with disease severity. Careful interpretation is necessary, as high ferritin levels can also occur in various other conditions, including renal failure, liver injury, infections, and hematologic malignancies in humans, as well as in dogs with lymphoma, HS, splenic hemangiosarcoma, and IMHA [57, 58]. Thus, elevated ferritin levels should not be used as a standalone marker for HLH (humans) or HPS (animals) but must be interpreted in the context of clinical symptoms (e.g., fever, splenomegaly) and other laboratory findings (e.g., cytopenias). Recognizing the high negative predictive value of a normal serum ferritin concentration can be useful for excluding HLH. Although elevated ferritin concentrations have been associated with histiocytic disorders in dogs, the usefulness of ferritin in the diagnosis of HPS in dogs has not been established [57, 58].

#### 4.3.2 Soluble Interleukin‐2 Receptor

The sIL‐2R (sCD25) is a significant biomarker for identifying both familial and secondary forms of HLH [59, 60]. This receptor is released from the surface of activated T lymphocytes, especially the CD8+ T cell subset, during periods of intense immune stimulation. Although sCD25 is known for its high sensitivity in detecting HLH, it lacks specificity, meaning that elevated levels of this biomarker may be seen in various other illnesses. For instance, higher sCD25 levels can be associated with lymphomas and leukemias, disease states where the immune system responds aggressively to tumor cells or their byproducts, leading to an increase in sIL‐2R levels [59]. Consequently, while elevated sCD25 levels are a valuable indicator in the diagnostic process, they cannot alone confirm a diagnosis of HLH; rather, they contribute to a broader diagnostic picture. Clinically, effective screening criteria for HLH in humans include serum ferritin that exceeds 500 ng/mL and sCD25 above 2400 U/mL [25, 55]. However, the specificity of these tests is significantly enhanced when sCD25 rises above 10 000 U/mL, especially if serum ferritin is concurrently increased above 5200 ng/mL [61]. Interpretation of both sCD25 and ferritin levels must always be contextualized with clinical assessments and other laboratory results to provide a comprehensive understanding of the patient's condition and to guide the diagnosis accurately.

A radioimmunoassay (RIA) for sCD25 has been developed for use in dogs, but this marker has not been systematically evaluated in canine patients with suspected HPS [62]. sCD25 may provide supportive evidence of immune activation in canine HPS; however, its diagnostic utility in this context remains uncertain. Serum sCD25 concentrations have been reported to be significantly increased in dogs with various forms of lymphoma [63], and because HPS in dogs is frequently secondary to lymphoma, increased sCD25 may reflect underlying neoplasia rather than HLH‐specific immune dysregulation. Nevertheless, the role of sCD25 as an ancillary diagnostic marker for HPS in dogs remains undetermined.

#### 4.3.3 NK Cell Activity

The evaluation of NK cell activity in people is a valuable diagnostic tool for distinguishing fHLH from sHLH [64, 65]. In fHLH, NK cell activity is often profoundly impaired or absent due to a mutation in genes critical for cytotoxic function. Thus, persistently low or absent NK cell activity, especially in the absence of an obvious trigger, raises suspicion for fHLH and should prompt further genetic investigation. This contrasts with sHLH, which is often associated with variable NK cell activity. NK cell function in patients with sHLH may be diminished due to immune exhaustion or cytokine‐driven suppression, but the impairment is typically less pronounced than in fHLH. If genetic changes are present in patients with sHLH, they contribute less directly to the pathogenesis and are frequently heterozygous mutations or polymorphisms in HLH‐related genes. Assessing NK cell activity involves specialized tests that measure the ability of NK cells to kill target cells, typically using in vitro assays such as flow cytometry‐based and cytotoxicity assays. In addition, newer tests for perforin expression and CD107a upregulation offer more sensitive options for assessing cytotoxic immune function and diagnosing primary HLH [66]. Research on the cytotoxic activity of NK cells in dogs is limited, with most studies conducted in research settings and not readily accessible for clinical use [67].

#### 4.3.4 Hypertriglyceridemia and Hypofibrinogenemia

Hypertriglyceridemia and hypofibrinogenemia are characteristic findings in HLH [68, 69]. Hypertriglyceridemia occurs due to increased cytokine‐driven lipolysis and reduced clearance of triglycerides by the liver, mediated in part by TNF‐α and other pro‐inflammatory cytokines. Hypofibrinogenemia results from excessive fibrinolysis and liver dysfunction, both driven by the same cytokine storm. Activated macrophages and tissue damage also promote the consumption of fibrinogen, contributing to decreased levels. These abnormalities are part of the diagnostic criteria for HLH and help differentiate it from other hyperinflammatory syndromes [25].

#### 4.3.5 Elevated Cytokines

Elevated inflammatory cytokines, particularly IFN‐γ, TNF‐α, and IL‐6, further support the diagnosis of HLH in humans. IFN‐γ, produced primarily by activated CTLs and NK cells, is a key driver of macrophage activation and hemophagocytosis [1, 70]. IFN‐γ also stimulates the release of downstream cytokines, amplifying inflammation. TNF‐α promotes tissue destruction, fever, and systemic inflammation, whereas IL‐6 contributes to hyperferritinemia, hypertriglyceridemia, and acute‐phase responses. The levels of these cytokines, particularly IFN‐γ, can help differentiate HLH from other hyperinflammatory conditions and correlate with disease severity. Emerging diagnostic tools, such as cytokine profiling or measurement of soluble cytokine receptors as previously described (e.g., sIL‐2R/sCD25), are increasingly being used to support an HLH diagnosis, especially in challenging cases where other criteria are ambiguous [71]. The measurements of IFN‐γ, TNF‐α, and IL‐6 are not included as standard cytokine tests for making a diagnosis based on the HLH‐2004 criteria [25].

#### 4.3.6 Hemophagocytosis

Hemophagocytosis is an important feature in the diagnosis of HLH [25]. In HLH, the dysregulated immune system leads to a hyperinflammatory response and infiltration of activated macrophages into various tissues. These activated macrophages engulf not only red blood cells, white blood cells, and platelets but also various cellular debris, resulting in cytopenias and contributing to the profound clinical manifestations of the disease. Although phagocytic activity is commonly observed in the bone marrow, it can also occur in other organs such as the spleen, liver, or lymph nodes. Hemophagocytosis is most readily detected in bone marrow cytology preparations, whereas in core biopsy samples, the phagocytic activity may be focal and harder to identify. Conversely, immunohistochemical markers can enhance the detection of histiocytic populations in the marrow core, allowing for a more precise definition of their distribution and phagocytic activity. It is important to note that the presence of hemophagocytosis is not essential for diagnosing HLH, and its absence does not exclude the diagnosis [25]. Further information on the role of hemophagocytosis in diagnosing HLH can be found in the subsequent section (Diagnosis of HLH—Bone Marrow Criteria).

#### 4.3.7 Immunohistochemistry for Histiocytic Cells in Bone Marrow

In normal bone marrow, histiocytes are typically inconspicuous and randomly distributed throughout the marrow space. They often localize within erythropoietic colonies, functioning as central macrophages or nurse cells [72]. Immunohistochemical markers such as CD11d, Iba1, and others [73, 74] are useful for identifying histiocytes in formalin‐fixed tissues of dogs and evaluating their distribution and morphologic features [75, 76]. These characteristics can provide valuable insights into the underlying disease process. For example, an increased number of macrophages diffusely scattered throughout the marrow or forming small clusters is suggestive of a reactive process, such as HLH [72]. In such cases, histiocytic cells typically appear well‐differentiated with minimal atypia, preserve the bone marrow architecture, and are often plump with foamy cytoplasm. Localized clusters or nodular aggregates of histiocytes are uncommon in HLH, but this pattern may suggest granulomatous disease or a localized infection. Although the patterns of histiocytic infiltration in human bone marrow have been more extensively characterized, comparable systematic evaluations distinguishing reactive and neoplastic histiocytic infiltrates in the bone marrow of dogs and cats are currently lacking.

In cases of HS, the bone marrow is often infiltrated by large, atypical histiocytic cells that can disrupt normal hematopoiesis and architecture. It is important to note that malignant histiocytes may exhibit only minimal morphologic atypia, and histiocytes in reactive conditions can sometimes display cellular atypia (Figure 3). Abnormal distribution patterns, such as widespread interstitial or intra‐sinusoidal accumulation of histiocytes, can also indicate a malignant population [72]. Although hemophagocytosis is uncommon in the dendritic cell subtype of HS involving the bone marrow, significant phagocytic activity can occur in the macrophagic subtype, complicating differentiation from HPS in animals [74]. In dogs, hemophagocytic histiocytic sarcomas (HHS) in the bone marrow express a CD11d phenotype, whereas dendritic malignancies are typically negative for this marker [73, 74]. Both malignant dendritic cells and their non‐malignant macrophagic counterparts are positive for histiocytic markers such as Iba1 in dogs and cats [76]. Therefore, interpreting morphologic findings and immunophenotypic markers in the context of clinical presentation, cytologic features, and molecular data are essential to distinguish between reactive conditions and neoplastic or infectious causes of marrow abnormalities.

**FIGURE 3:**
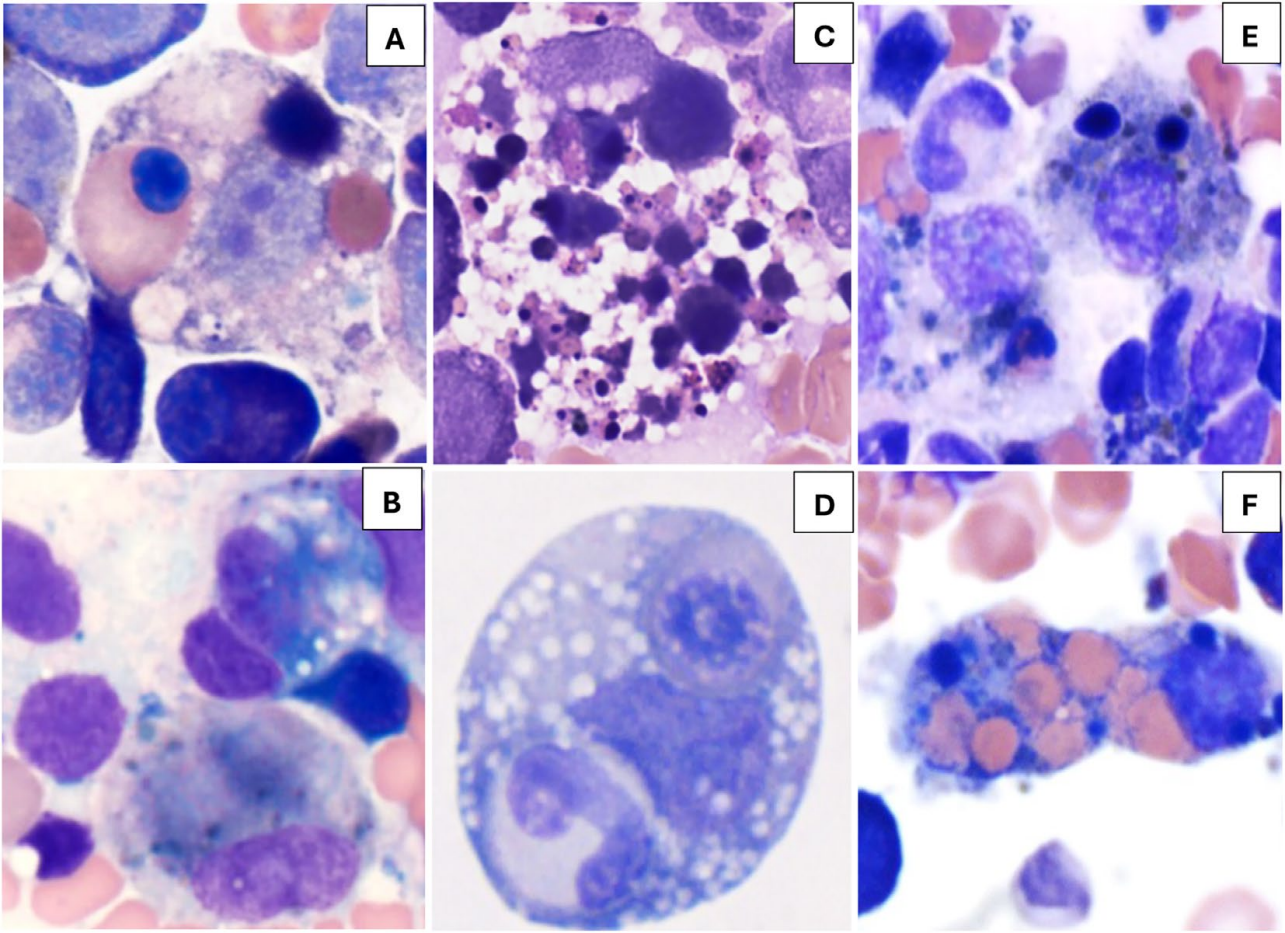
Hemophagocytosis in bone marrow aspirates is associated with various neoplastic and non‐neoplastic diseases. (A, B) neoplastic histiocytes from two dogs with histiocytic sarcoma; (C, D) reactive histiocytes identified in two dogs with hemophagocytic syndrome; and (E, F) reactive histiocytes from dogs with drug‐induced and post‐transfusion‐associated phagocytic activity (almost exclusively erythrophagia). The key takeaway is that morphology (degree of atypia and type of phagocytic activity) alone cannot reliably differentiate between malignant and reactive histiocytes (Wright's stain).

#### 4.3.8 Immunohistochemistry and Distribution of Lymphoid Cells in Bone Marrow

CD3 and CD20 staining in bone marrow biopsies are critical for differentiating between reactive and neoplastic lymphoid infiltrates, particularly in the context of diagnosing HLH and HPS. This distinction is essential because HLH and HPS can arise as a secondary process to lymphoid malignancies in humans and veterinary species [6, 27, 28, 29, 31, 39, 77]. By identifying T‐cell (CD3 positive) and B‐cell (CD20 positive) populations and assessing their distribution, these markers help clarify whether the underlying pathology is a reactive immune response or a malignant process. Further, advanced diagnostics in dogs and cats, such as PCR for Antigen Receptor Rearrangements (PARR) of the bone marrow aspirate, should be pursued when clinical suspicion points to the possibility of an underlying malignancy driving the development of the syndrome [78, 79].

In humans, reactive lymphoid infiltrates, including those associated with HLH, are typically polyclonal and localized or form small aggregates, whereas neoplastic infiltrates often exhibit monoclonality and a more diffuse distribution. The lymphoid cells in reactive conditions are morphologically normal, with no significant atypia [80, 81]. In neoplastic conditions like lymphoma, the lymphoid cells often form diffuse, poorly circumscribed infiltrates, which can disrupt or obliterate the normal architecture of the bone marrow. The neoplastic cells often exhibit abnormal morphology, such as increased size, irregular nuclear outline, and prominent nucleoli. Marrow involvement of the neoplastic lymphocytes may sometimes show a paratrabecular or an interstitial pattern of infiltrates [82, 83, 84]. Similar distributions can also be seen in veterinary species, where reactive lymphoid infiltrates tend to be focal and polyclonal, whereas neoplastic infiltrates are more often diffuse and monoclonal.

## 5 Diagnosis of HLH (The Preponderance of the Evidence)

### 5.1 HLH‐2004 Diagnostic Criteria

The clinical features of HLH, including fever, splenomegaly, and cytopenias, share similarities with various other conditions like sepsis, systemic inflammatory response syndrome, malignancies, autoimmune disorders, and infections such as tuberculosis or viral illnesses [25, 85, 86]. This overlap poses a challenge in making a diagnosis of the syndrome, highlighting the necessity of combining clinical observations with both routine and specialized laboratory tests to differentiate HLH from these other conditions. The most referenced guidelines for diagnosing HLH were established in 2004 (e.g., HLH‐2004 criteria). Although initially created for fHLH in pediatric trials, these criteria have become widely applicable for supporting the diagnosis of secondary sHLH [25, 86].

According to the HLH‐2004 criteria, the diagnosis requires at least two out of three clinical manifestations: fever, splenomegaly, and hemophagocytosis [25]. Additionally, it mandates three out of five specific laboratory abnormalities, which include hyperferritinemia, elevated triglycerides, low fibrinogen levels, increased sCD25 (an indication of immune activation), and cytopenia affecting two or more cell lines. These criteria are essential for pinpointing potential cases of fHLH that might need additional genetic testing, and they play a crucial role in distinguishing it from sHLH. A definitive diagnosis of fHLH can be made by detecting pathogenic mutations in critical genes for cytotoxic immune function, such as PRF1, UNC13D, or STXBP2. Given that hemophagocytosis and certain laboratory markers (e.g., serum ferritin, triglycerides) may overlap with other hyperinflammatory or infectious conditions, careful analysis of the criteria alongside molecular testing is essential for confirming fHLH [25, 86].

Although a significantly elevated serum ferritin (e.g., > 10 000 ng/mL) suggests a diagnosis of HLH (whether fHLH or sHLH), it is not definitive on its own [53]. Conversely, a normal ferritin concentration strongly suggests alternative diagnoses. Hemophagocytosis is the only criterion in the HLH‐2004 that does not have a specific threshold value [25]. Although its presence supports an HLH diagnosis, it cannot independently confirm the syndrome. Studies have shown that many HLH patients do not exhibit hemophagocytosis on initial bone marrow biopsies [87]. If clinical concern persists, a repeat bone marrow biopsy should be conducted, or biopsies of other affected tissues, such as the spleen, liver, or lymph nodes, can be performed where hemophagocytosis might be more apparent [87]. Correct evaluation of clinical signs, laboratory markers, and genetic testing as outlined by the HLH‐2004 criteria is vital for accurately diagnosing HLH and distinguishing it from other overlapping conditions [86]. Currently, there is no established consensus on the guidelines for diagnosing HPS in dogs and cats. There is a clear need for relevant criteria to establish a diagnosis of HPS and the necessary thresholds to exclude conditions that mimic it in these animals.

### 5.2 HScore

The HScore is a diagnostic tool developed to estimate the probability of HLH, particularly sHLH in adults, using clinical, laboratory, and bone marrow aspirate findings [88]. It was created to address challenges in diagnosing HLH in adults with complex presentations, as existing criteria like HLH‐2004 were primarily designed for pediatric populations and fHLH. Unlike HLH‐2004, which requires five of eight criteria, including specialized tests like NK cell activity and sCD25 levels, the HScore focuses on parameters more readily available in routine clinical settings [88]. These include clinical features (e.g., fever, splenomegaly), laboratory results (e.g., cytopenias, hyperferritinemia, hypofibrinogenemia), and the presence of hemophagocytosis on tissue biopsy. This scoring algorithm also incorporates underlying immune suppression and liver dysfunction, which are not in HLH‐2004 [25]. Weights are assigned to these variables, yielding a total score that corresponds to the likelihood of HLH. An online calculator simplifies its use and is available at HScore Calculator [89].

In human medicine, the HScore is particularly useful in cases with overlapping or equivocal clinical and laboratory findings and serves as a complement to clinical judgment when deciding whether to initiate therapy while diagnostic confirmation is ongoing [88]. Importantly, the composite nature of the HScore helps distinguish clinically significant HLH from secondary or incidental hemophagocytic activity, such as that observed following recent blood transfusion, by integrating the severity of cytopenias, degree of hyperferritinemia, extent of organomegaly, and systemic inflammatory features rather than relying on marrow hemophagocytosis alone. Although the HScore is not designed to distinguish HLH from neoplastic histiocytic disease [88], its structure can support that distinction when used in conjunction to features such as histiocytic atypia, architectural effacement of involved tissues, and increased proliferative activity—features not captured by the HScore algorithm or HLH‐2004 Diagnostic Criteria [25, 88, 90].

Application of the HScore in veterinary species remains limited due to the lack of validated thresholds for assigning scores to individual criteria. A comparison of the HLH‐2004 and HScore criteria is summarized in Table 1 [25, 88]. Although these frameworks may assist in structured assessment, they have not been validated in veterinary species. Therefore, diagnosis of HPS in dogs and cats requires comprehensive clinicopathologic correlation using a systematic, stepwise approach, as outlined below:

**TABLE 1:** Diagnosis of HLH based on HLH‐2004 criteria and H‐score.

	Primary HLH HLH‐2004 Criteria	Secondary HLH & MAS H‐score 2014
Target population	Primary HLH	Adults HLH
**Clinical Features** Fever	+	< 39.4°C (0), 38.4‐39.4°C (33), > 39.4°C (49)
Hepatomegaly Splenomegaly	+	Neither (0), Either/Or (23), Both (38)
Immunosuppression	**NA**	No (0), Yes (18)
**Laboratory Features** Cytopenia > 2 lineages	Hemoglobin < 9 mg/dL; Neutrophils < 1 K/μL; Platelets < 100 K/μL	1 lineage (0), 2 lineages (24), 3 lineages (34)
Ferritin	≥ 500 μg/L	< 2000 μg/L (0), 2000‐6000 μg/L (35), > 6000 μg/L (50)
Hypertriglyceridemia Hypofibrinogenemia	≥ 3 mmol/L ≤ 1.5 g/L	> 2.5 mmol/L (0), < 2.5 mmol/L (30) > 2.5 g/dL (0), < 2.5 g/dL (30)
Aspartate aminotransferase (AST)	**NA**	< 30 U/L (0), > 30 U/L (19)
Soluble CD25	≥ 2400 U/mL	**NA**
Low or absent natural killer (NK) activity	+	**NA**
Hemophagocytosis	+	No (0), Yes (35)
**Fulfillment of criteria**	**5/8 criteria**	**Score > 169 suggesting a high probability of HLH**

*Note:* The diagnostic threshold for HLH‐2004 requires either (i) a molecular diagnosis based on the presence of a known HLH‐causing genetic mutation or (ii) meeting five of eight clinical and laboratory criteria, whereas an H‐score diagnosis is based on weighted variables (score shown in parentheses) with a cumulative score > 169 suggesting a high probability of HLH. A web‐based calculator for the H‐score is available at the link provided. The diagnosis of HPS in dogs and cats relies heavily on only two criteria—cytopenias and hemophagocytosis—whereas other clinical and laboratory features included in diagnostic schemes are applied inconsistently in veterinary species. +, feature is present; NA, not applicable.

#### 5.2.1 Clinical and Hematologic Assessment

Document persistent cytopenias (bi‐ or pancytopenia) and systematically assess clinical findings for evidence of an underlying inflammatory or neoplastic process.

#### 5.2.2 Bone Marrow Evaluation

Perform morphologic assessment of aspirate and core biopsy specimens, with particular attention to hemophagocytosis, overall histiocyte burden, and architectural disruption. Immunohistochemical characterization of histiocytes is recommended to assess distribution and phenotype.

#### 5.2.3 Exclusion of Reactive Causes

Systematically evaluate for conditions associated with secondary hemophagocytosis, including severe systemic inflammatory disease or sepsis, immune‐mediated cytopenias (e.g., IMHA, PIMA, ITP), infection, severe drug reactions, and post‐transfusion responses.

#### 5.2.4 Ancillary Testing

When indicated, incorporate molecular testing (e.g., PARR) and additional immunophenotypic studies to distinguish reactive histiocytic proliferation from malignant histiocytic disorders.

### 5.3 Bone Marrow Criteria

Recent studies indicate that specific morphologic characteristics identified in bone marrow aspirates are useful for diagnosing HLH and distinguishing it from other diseases [87, 90, 91]. A principal diagnostic marker for HLH is the presence of hemophagocytosis, a process in which activated macrophages consume hematopoietic elements, including red blood cells, white blood cells, and platelets. Patients with HLH typically show significantly higher numbers of phagocytized hematopoietic precursors from various lineages compared to those with other conditions [87].

The accuracy of diagnosing HLH can be improved by applying quantitative thresholds for the types of cells that are phagocytized, whether individually or in combination [87]. For instance, nearly 100% certainty of an HLH diagnosis in human patients is achieved when at least one hemophagocyte contains a granulocyte, two or more hemophagocytes contain nucleated RBCs, and one hemophagocyte contains lymphocytes. The presence of multiple nucleated cells within a single hemophagocyte is also a strong indicator of HLH in symptomatic patients. This study also found that individuals without hemophagocytes ingesting granulocytes had only a 3% likelihood of having HLH [87].

It is important to note that hemophagocytosis, whereas a hallmark feature of HLH, can also occur infrequently in other conditions such as severe infections, autoimmune diseases, and certain malignancies [7]. Moreover, several studies have emphasized that the phagocytosis of non‐nucleated RBCs alone is a nonspecific finding and cannot independently confirm HLH [87]. Diagnosing HLH requires a comprehensive evaluation of the patient within the appropriate clinical context, combining clinical, laboratory, and histopathologic data. Detailed microscopic examination of bone marrow aspirate smears is crucial for identifying hemophagocytosis but must be interpreted alongside other diagnostic criteria to avoid misdiagnosis and ensure accurate identification of HLH [87, 91].

### 5.4 Case Illustrations of Histiocytic Disorders in Dogs Aligned With HLH‐2004–Style Diagnostic Criteria [25]

Three bone marrow cases are presented to illustrate the spectrum of histiocytic disorders encountered in dogs, including two cases of reactive HPS and one representing HHS. The goal is to demonstrate how bone marrow morphology, distribution of histiocytes, and ancillary testing can be interpreted within a framework analogous to the HLH‐2004 diagnostic criteria (Figures 4, 5, 6). Although not all HLH‐2004 criteria are routinely evaluated or validated in veterinary patients, applying this structured approach provides a useful conceptual scaffold for distinguishing reactive from neoplastic hemophagocytic disorders. Across these cases, several HLH‐2004 criteria were fulfilled or strongly supported, including cytopenias affecting ≥ 2 lineages, hemophagocytosis in bone marrow, and evidence of an underlying triggering condition (malignancy). Clinical features such as fever and splenomegaly or hepatomegaly, along with other HLH‐2004 laboratory diagnostic criteria, were inconsistently reported; however, these findings remain essential components of the diagnostic assessment and should be evaluated as fully as possible when HPS is suspected.

### 5.5 Acute Myeloid Leukemia–Associated Hemophagocytic Syndrome

Figure 4 illustrates bone marrow findings from a 2‐year‐old Golden Retriever dog diagnosed with acute myeloid leukemia (AML)–associated HPS or malignancy‐associated HPS (M‐HPS) (courtesy of Dr. Hamideh Esmaeilzadeh). Diagnostic features consistent with HLH‐2004–style criteria included bi‐cytopenia (non‐regenerative anemia and thrombocytopenia) and hemophagocytosis of erythroid and myeloid precursors within the bone marrow [25].

**FIGURE 4:**
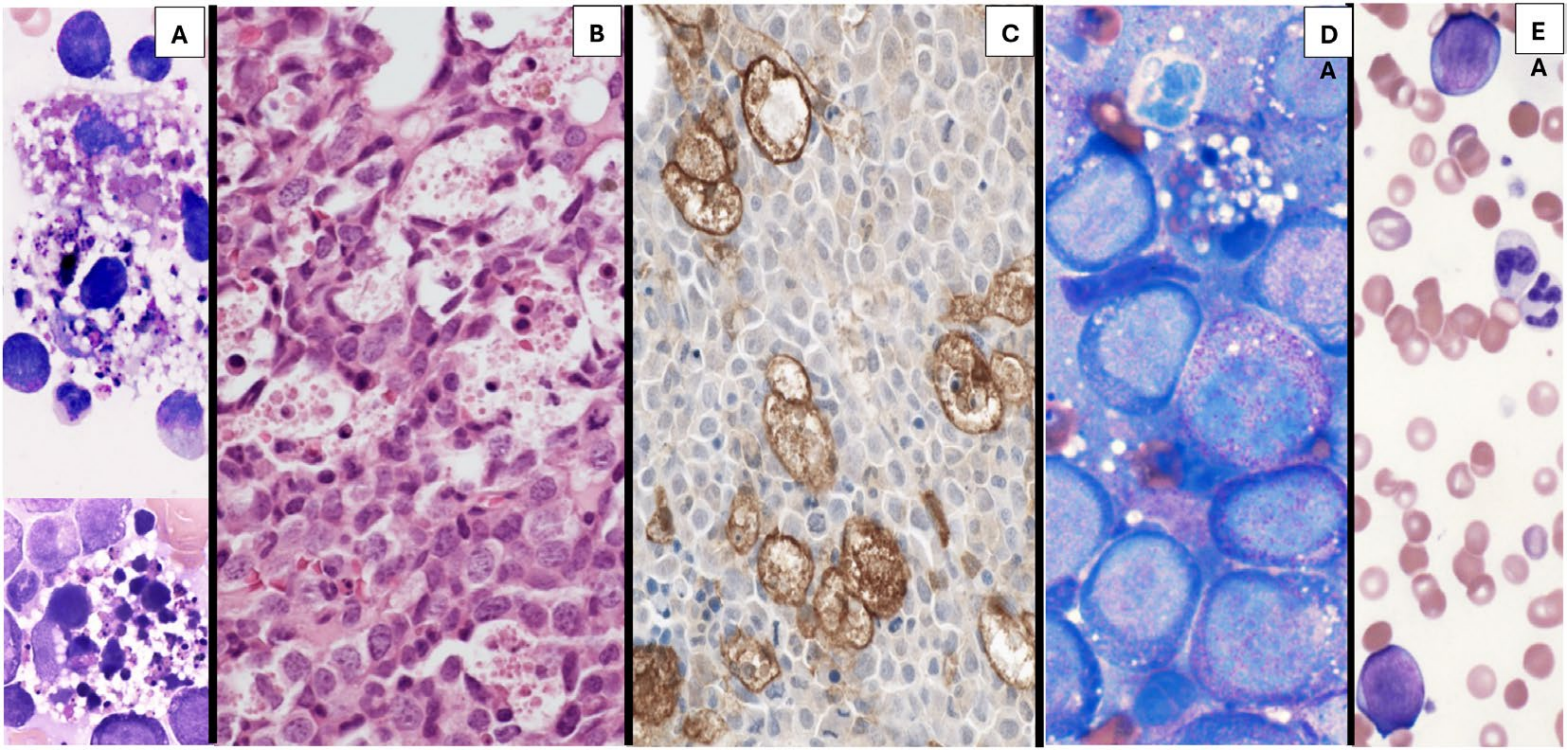
Bone marrow from 2‐year‐old Golden Retriever dog with an acute myeloid leukemia (AML) and malignancy‐associated hemophagocytic syndrome (M‐HPS), courtesy of Dr. Hamideh Esmaeilzadeh. (A) Bone marrow aspirate cytology shows a bland population of activated histiocytes/macrophages containing particulate cytoplasmic debris, as well as phagocytized erythroid and myeloid precursors. Wright stain, 1000×. (B) Bone marrow core histology shows individual and clustered reactive histiocytic cells amid predominantly immature hematopoietic cells, with > 20% blasts consistent with AML based on morphology and flow cytometry. H&E stain, 600×. (C) Immunohistochemistry of bone marrow core showing individual and small clumps of histiocytes positive for iba‐1 and intervening immature hematopoietic cells. Anti‐Iba‐1 and hematoxylin counterstain, 600×. (D) Lymph node fine‐needle aspirate showing abundant immature myeloid precursors, primarily myeloblasts and promyelocytes. Wright stain, ×1000. (E) Peripheral blood smear demonstrates approximately 10% blasts, reflecting the spillover of immature myeloid precursors into the circulation. Wright stain, ×600.

Bone marrow examination revealed a proliferation of bland, foamy macrophages comprising approximately 5% of all nucleated cells, which were distributed individually and in small clusters throughout the marrow spaces. These histiocytes exhibited exuberant hemophagocytosis yet caused minimal disruption of marrow architecture (spatial organization and structural framework of marrow are preserved), supporting a reactive histiocytic process. In contrast, the hematopoietic compartment demonstrated a markedly expanded mitotic pool of myeloid precursors, with > 20% myeloblasts, fulfilling criteria for AML and providing a clear secondary trigger, analogous to malignancy‐associated HLH in humans [7].

Disease progression was further supported by findings 1 month after initial presentation, when a lymph node aspirate revealed an infiltrate of myeloid precursors with 38% myeloblasts, accompanied by circulating blasts in the peripheral blood. Flow cytometry of the node demonstrated a large population of hematopoietic cells, with 45% CD45^+^ cells and a markedly expanded CD34^+^ fraction (20%), far exceeding the normal bone marrow range of approximately 1 to 3% CD34^+^ cells. These findings indicate a significant increase in immature progenitor cells. PARR analysis showed polyclonal T‐cell receptor and immunoglobulin gene rearrangements, excluding a lymphoid neoplasm. Collectively, the morphologic, immunophenotypic, and molecular findings support an expanded blast population consistent with AML and establish this case as secondary HPS driven by AML, interpreted within a diagnostic framework analogous to the HLH‐2004 criteria.

### 5.6 HPS Secondary to Large Granular Lymphocyte (LGL) Lymphoma/Leukemia

Figure 5 depicts HPS secondary to LGL lymphoma/leukemia in a 4.5‐year‐old female Old English Mastiff (courtesy of Dr. Mara Varvil). This case fulfilled multiple HLH‐2004–style criteria [25], including pancytopenia, bone marrow hemophagocytosis of multiple hematopoietic cell lineages, and identification of an underlying T‐cell malignancy as the inciting trigger.

**FIGURE 5:**
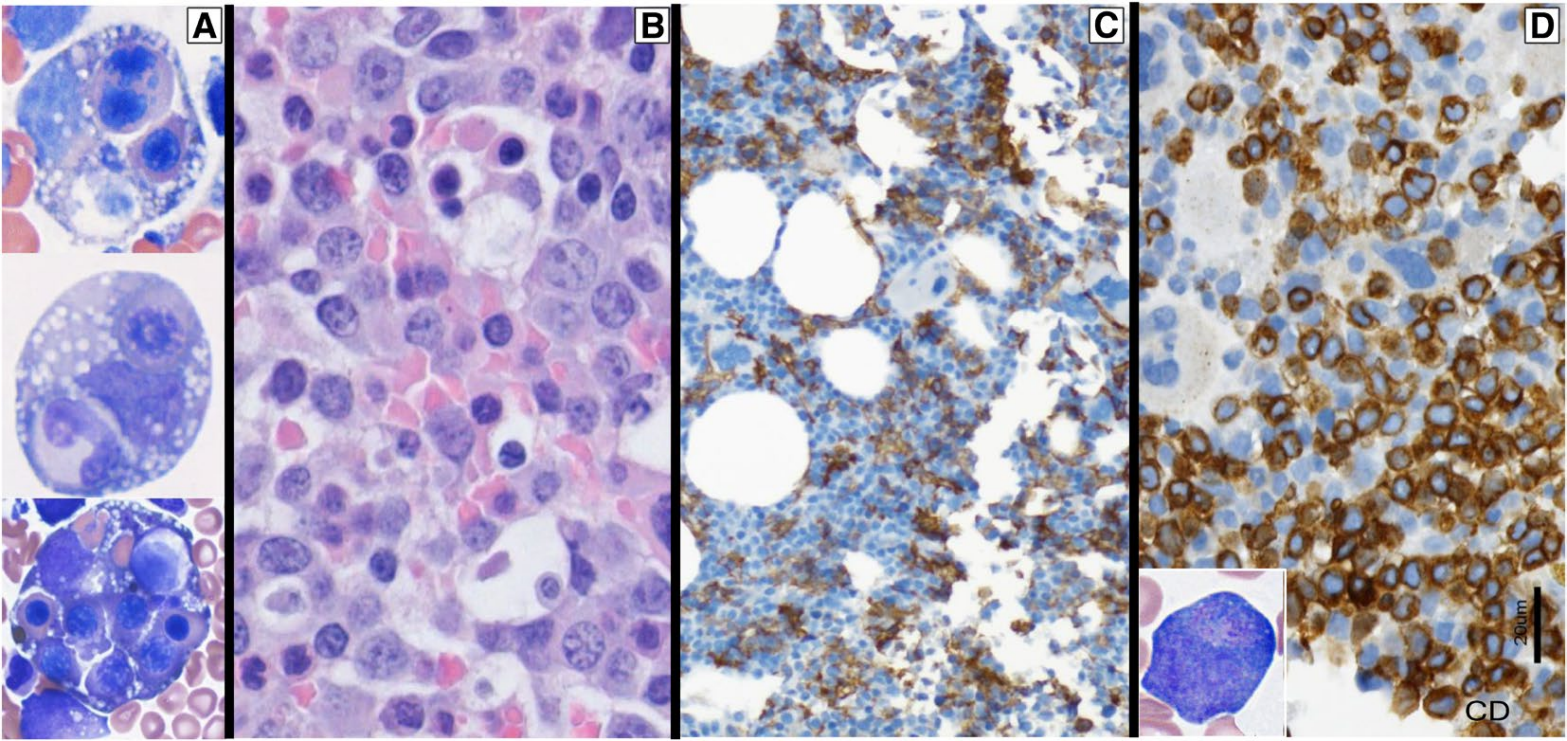
Bone marrow aspiration and core biopsies from a 4.5‐year‐old female Old English Mastiff dog with hemophagocytic syndrome associated with large granular lymphocyte (LGL) lymphoma/leukemia (M‐HPS), courtesy of Dr. Mara Varvil. (A) Histiocytes exhibiting hemophagocytosis of erythrocytes, rubricytes, and leukocytes. Wright stain, 1000×. (B) Bone marrow core histology also demonstrated hemophagocytosis by histiocytes, particularly within areas infiltrated by neoplastic lymphocytes. H&E, 600×. (C) CD11d‐positive histiocytes were increased in number and distributed both individually and in small aggregates among intervening hematopoietic cells. Anti‐CD11d and hematoxylin counterstain, 200×. (D) Expansion of CD3‐positive neoplastic T lymphocytes within the interstitium in multifocal areas. Anti‐CD3 and hematoxylin counterstain, 600×; inset LGL from bone marrow aspirate, Wright stain, ×1000.

Despite peripheral pancytopenia, the bone marrow was hypercellular. Minimally atypical, foamy macrophages comprised approximately 3.5% of all nucleated cells and exhibited hemophagocytosis of erythrocytes, rubricytes, and granulocytes. Special stains facilitated recognition of this expanded histiocytic population, which remained distributed individually or in small aggregates with minimal disruption of normal marrow architecture, consistent with a reactive process. In addition, routine H&E‐stained sections showed multifocal interstitial expansion by neoplastic round cells. A bone marrow aspirate cytology demonstrated that these cells contained packed, pinkish cytoplasmic granules and were morphologically consistent with LGLs. Immunohistochemistry confirmed the cells to be CD3‐positive T lymphocytes, and PARR analysis demonstrated a monoclonal T‐cell receptor gene rearrangement, confirming LGL lymphoma/leukemia [92]. The coexistence of a reactive hemophagocytic macrophage population with a distinct neoplastic lymphoid infiltrate supports classification of this case as secondary HPS, analogous to malignancy‐associated HLH under HLH‐2004 criteria.

### 5.7 Hemophagocytic Histiocytic Sarcoma (HHS)

Figure 6 illustrates bone marrow findings from a 7‐year‐old male Bernese Mountain Dog diagnosed with HHS. Although this case also fulfilled several HLH‐2004–style criteria [25], including bi‐cytopenia (regenerative anemia and thrombocytopenia) and marked hemophagocytosis, the neoplastic nature of the histiocytic population distinguishes it from reactive HPS.

**FIGURE 6:**
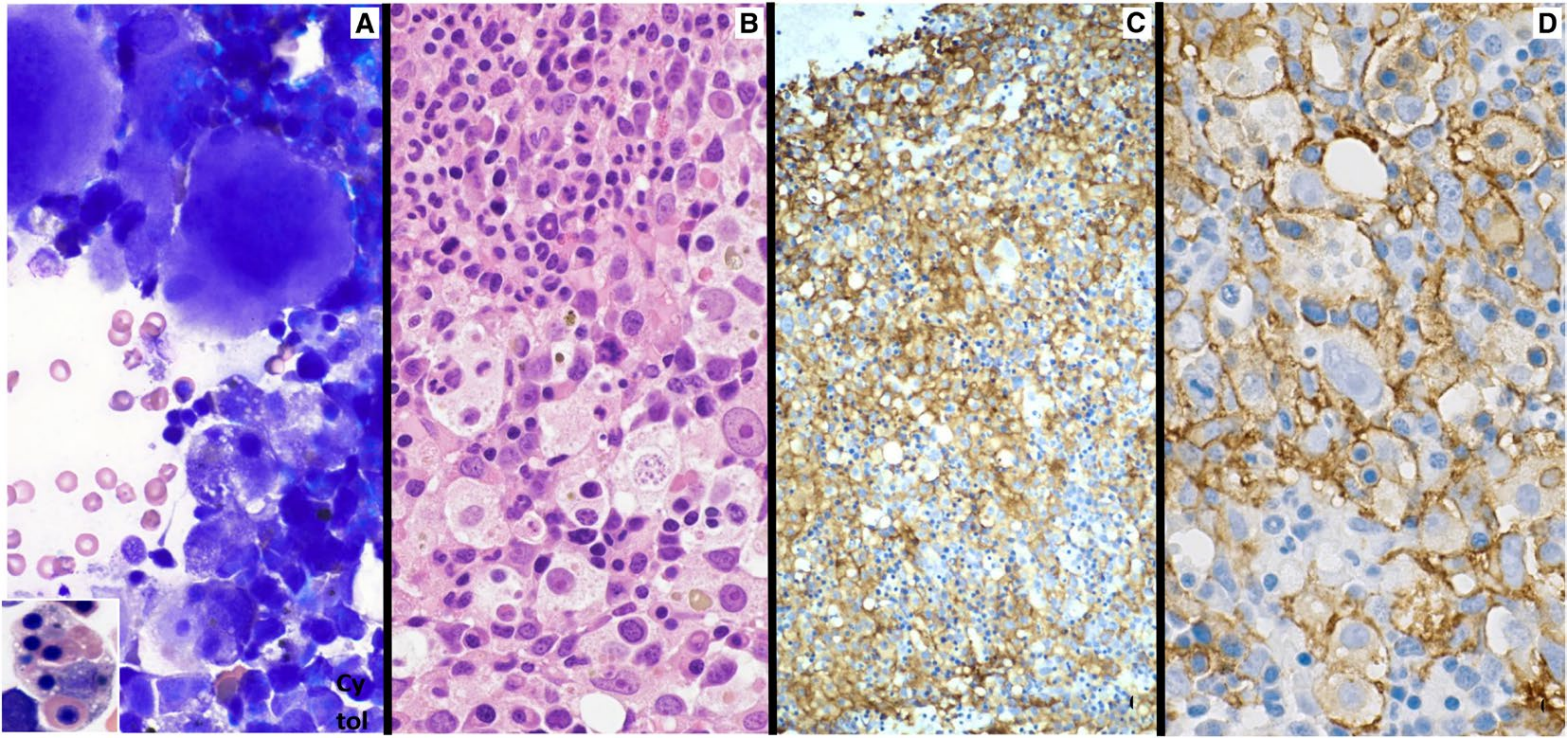
Bone marrow features of hemophagocytic histiocytic sarcoma (HHS) in a 7‐year‐old Bernese Mountain Dog. (A) Bone marrow aspiration cytology shows several phagocytic histiocytes (black arrows) adjacent to a bone marrow particle. Wright stain, 600×; Insert: Marked rubriphagia by a malignant histiocyte. Wright stain, ×1000. (B) Bone marrow core histology shows myeloid precursors in the upper portion and neoplastic histiocytes in the lower portion. H&E, 600×. (C) Bone marrow core biopsy showing a diffuse, widespread infiltrate of CD11d‐positive neoplastic histiocytes and multifocal effacement of hematopoietic tissue. CD11d IHC with hematoxylin counterstain, ×200. (D) Markedly anaplastic histiocytes exhibit marked phagocytic activity; note the prominent, large nucleoli. CD11d and hematoxylin counterstain, 600×.

Bone marrow cytology demonstrated hemophagocytic histiocytes comprising approximately 6% of the absolute nucleated cell count, with predominant rubriphagia and occasional leukophagocytosis. Unlike the HPS cases, histologic evaluation showed marked histiocytic atypia and diffuse architectural disruption, characterized by filling of paratrabecular and multifocal interstitial regions by neoplastic histiocytes. Immunohistochemistry showed strong CD11d positivity, confirming macrophage lineage and supporting a diagnosis of HHS [75]. When considered alongside breed predisposition, regenerative anemia, and documented invasion of hepatic and splenic tissues by neoplastic histiocytes, the marrow findings in this case are most consistent with primary hemophagocytic malignancy, rather than secondary HPS. Thus, despite overlapping HLH‐2004 features, the distribution, cytologic atypia, and architectural effacement clearly separate HHS from reactive hemophagocytic syndromes [25, 75, 86].

### 5.8 Integrative Interpretation

Across all cases, application of an HLH‐2004–style diagnostic framework underscored the importance of integrating peripheral cytopenias, bone marrow hemophagocytic activity, and identification of an inciting trigger with careful evaluation of histiocytic morphology and distribution. Reactive hemophagocytic syndrome (HPS) was generally characterized by localized or loosely aggregated histiocytes within a preserved marrow architecture. In contrast, HHS more often demonstrated diffuse infiltration, cytologic atypia, and architectural effacement.

Importantly, these morphologic features are not consistently discriminatory. Reactive histiocytic proliferations may be extensive, diffusely interstitial, and exhibit cytologic atypia, at times mimicking neoplasia. Conversely, histiocytic malignancies may display relatively bland cytologic features with minimal pleomorphism. Accordingly, definitive classification requires interpretation within the broader clinical and clinicopathologic context rather than reliance on morphology alone.

Ancillary diagnostics—including immunohistochemistry, flow cytometric immunophenotyping, and molecular assays such as PARR—were essential for confirming histiocytic lineage and identifying occult lymphoid neoplasia when present. Additional laboratory parameters incorporated into the HLH‐2004 criteria, including serum ferritin and sIL‐2R/sCD25—the latter validated in dogs—were not consistently available in these cases. When accessible, such markers may provide further evidence of immune dysregulation and strengthen differentiation between reactive and neoplastic hemophagocytic disorders [63].

### 5.9 Proposal for Histiocytic Study Group: Prospective Multi‐Institutional Research

A multi‐institutional study is proposed to evaluate and validate diagnostic criteria for HPS in dogs, distinguishing it from other reactive and neoplastic histiocytic/macrophagic disorders (Figure 7). The objective of this study is to assess and validate diagnostic criteria for HPS in dogs and determine whether these criteria effectively distinguish HPS from other reactive and neoplastic histiocytic/macrophagic disorders. Specific aims would include (i) validation of HLH‐2004 criteria in canine cases, (ii) adaptation and assessment of the H‐score for dogs; and (iii) analysis of bone marrow topography, utilization of immunohistochemistry to identify histiocytic and lymphoid cell populations, and incorporation of molecular markers to refine the diagnosis. Morphology of macrophages and quantitative thresholds for hemophagocytosis would also be analyzed. To rule out the possibility of an underlying neoplasm or HLH‐related genetic mutation, PARR and molecular testing will be conducted as appropriate. Statistical methods will determine the sensitivity, specificity, and predictive value of proposed criteria to define thresholds and optimize diagnostic accuracy. The aim of the study will be to provide validated diagnostic criteria tailored for canine HPS, offering a more precise differentiation from other histiocytic and macrophagic disorders.

**FIGURE 7:**
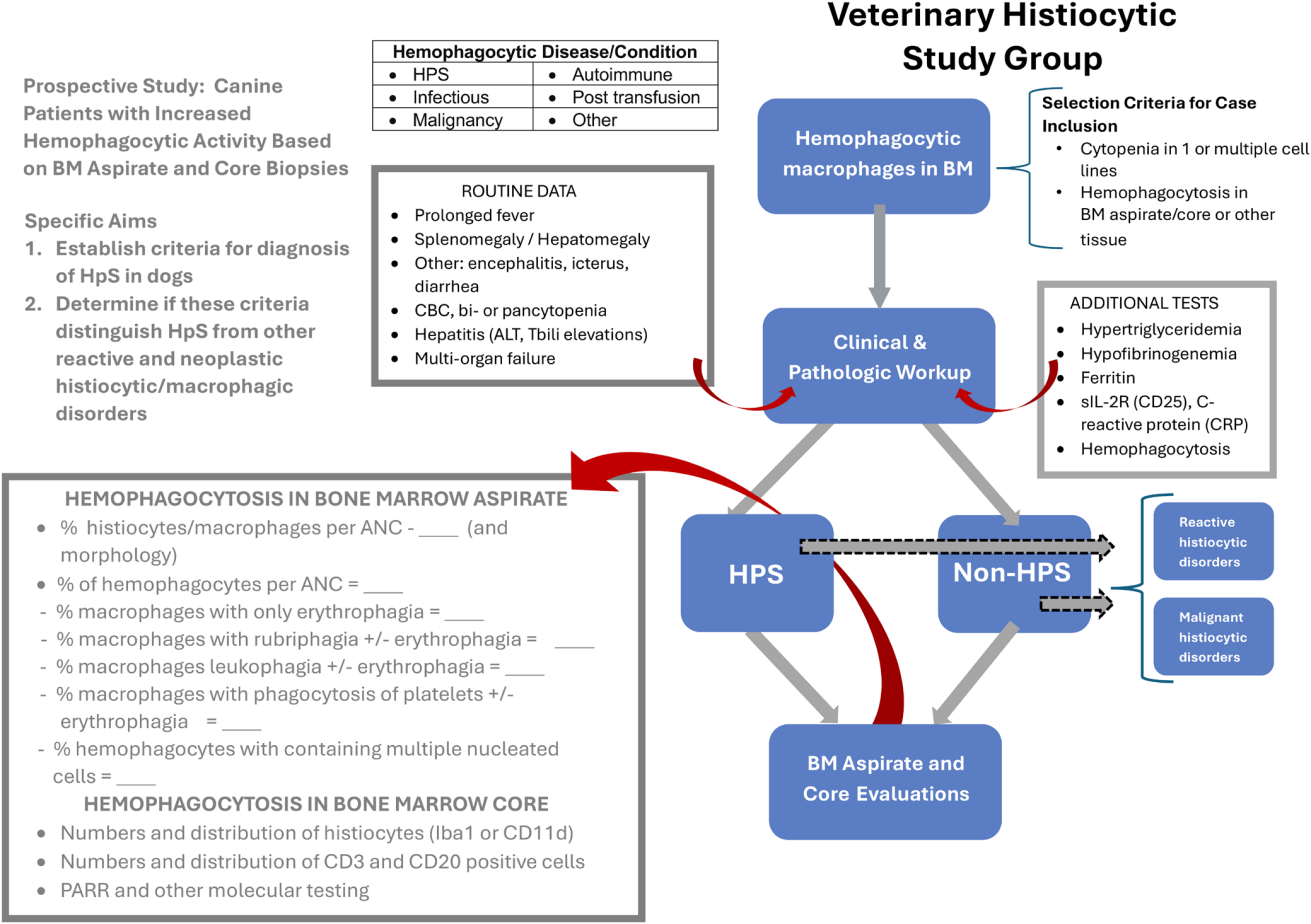
Flow chart for proposed prospective multi‐institutional study. To include patients with a wide variety of histiocytic conditions, case selection is based on (i) cytopenia in 1 or more cell lines; and (ii) hemophagocytosis in BM aspirate/core or other tissues. Cases are categorized as HPS or non‐HPS based on clinical and pathologic criteria, with the HLH‐2004 criteria serving as the foundational framework. BM aspirate and core biopsy specimens are evaluated in a blinded manner for morphologic features, phagocytic activity, and particularly for the number and distribution of histiocytes, using H&E and IHC staining (large red arrow). These findings are then correlated with patient classification data (dotted arrow) to establish a definitive diagnosis of HPS or non‐HPS. PARR and other molecular and IHC assays, are performed as indicated to classify non‐HPS cases as reactive or malignant histiocytic disorders. ANC, all nucleated cells; BM, bone marrow; HPS, hemophagocytic syndrome; IHC, immunohistochemistry; PARR, polymerase chain reaction for antigen receptor rearrangements.

## 6 Conclusions

HPS/HLH is a severe hyperinflammatory syndrome driven by dysregulated immune activation, leading to excessive cytokine release, immune cell proliferation, and organ damage. Its pathophysiology is characterized by impaired cytotoxic function of NK cells and CTLs, often resulting from genetic mutations in fHLH or triggered by infections, malignancies, or autoimmune disorders in sHLH. Diagnosis remains challenging, requiring a combination of clinical, laboratory, and histopathologic findings. Tools like the HLH‐2004 diagnostic criteria and HScore offer structured approaches, but bone marrow examination and molecular testing, including evaluation of hemophagocytosis and clonal lymphocyte populations, are vital for distinguishing HPS from mimicking conditions. The proposed study suggests an initial effort to establish diagnostic criteria and determine appropriate thresholds for differentiating HPS from other histiocytic disorders in dogs, with the potential for future application to cats. This represents a crucial step toward enabling future research focused on evaluating treatment strategies and improving outcomes for this life‐threatening syndrome.

## Conflicts of Interest

The author declares no conflicts of interest.

## Data Availability

The author has nothing to report.
